# The Use of Mat Golds

**Published:** 1902-10-15

**Authors:** 


### THE USE OF MAT GOLDS.

In a clinic before the Missouri State Dental Society, Dr. Hetrick made a clinic in filling teeth with Vernon's gold and the following is an abstract of some parts of the discussion.

Dr. S. S. Noble said:—In the first place, we want to remember that in this gold we have a pure gold, it is simply a question of whether we condense it properly if our work is done well or ill.

The last thing I did before leaving my office was to build an incisor tooth that had a triangular-shaped cavity. The cutting edge was built up half its width, and contoured with Vernon's gold. I have as much confidence in its lasting qualities as I ever did in any gold that I have used, and it has nearly or quite spoiled me for using any other gold, because I am accustomed to it, and am so thoroughly satisfied with its working qualities.

This matter of proper annealing is the keynote in handling this gold. I never saw a gold that would stand as much of it and not be injured by it. Dr. Hetrick said he would not advise annealing in an open flame. I think a wood alcohol flame will even ruin it for good work, yet I do not hesitate to pass it into a Bunsen burner flame until it is red-hot; at the same time, that is not the best way to treat it; but if my Bunsen burner is behaving badly, and it does not heat the mica well, if I am annealing, in order that I may have a good filling, I pass the gold through the flame and heat it more thoroughly. In annealing it properly over the mica, it works smoothly, does not work harsh because you do not melt the crystals. I am satisfied, if you anneal it in a Bunsen flame, it will not work as readily. Another gentleman told me he had passed it through alcohol flame, and it spoiled it quite. I have not had that experience with it, and cannot say.

With regard to the waste, there is no waste at all; if the gold is properly annealed, it will hold particle to particle. You can imagine almost you are using sheet gold. If you do drop a particle of the gold, when you pick up your next piece, touch it to that particle and it attaches to it as if it were a magnet.

There is another danger in using Vernon's gold, in filling small cavities, you will bridge your cavity. We use larger points in using this gold, than in the ordinary sheet gold, because you are liable to puncture it before it is condensed. In placing the gold in the cavity, use as large a point as your cavity will permit, and not bridge at any part, and then use a finer point to complete the filling; otherwise you will cut right through the gold and chop it before you get started. But it will not rock in any event. You will forget that gold ever did rock after you have used it a little while. After you have partially condensed, use a plugger with a point small enough to enter the groove or whatever you constructed as a retainer. The point should always be small enough to come in contact with the form of your cavity in completing the work, but first use a large point—there are one or two made for the purpose.

In beginning, as Dr. Hetrick suggests, be sure and cut the particles of gold so that they will not be larger than the cavity and will not double up. Cut them the full size of the cavity if you can, so that a slab of it reaches over the whole face of the cavity that you wish to fill. It will surprise you how nicely it will work. I condense with an automatic plugger. The first instructions that came out with this gold advised the use of hand-pressure entirely, but my courage was not equal to the occasion and I used an automatic plugger more satisfactorily. I use a point and foot-plugger, having the two conveniently at hand, and other point after I get the filling started. In small cavities you are likely to have failures because you are apt to bridge your cavity, it is necessary to use great care to secure a proper adaptation.

Dr. W. R. Cone, of Hutchinson. I want to say a few words in regard to Vernon's gold. I have had a practical demonstration of the value of the use of this gold to-day. Dr. Hetrick put in two large fillings for me in the clinic, in less time than it would take to put in one filling, I believe, with any other gold, and the ease with which he operated this gold was a revelation to me.

There is one thing which Dr. Hetrick does that I believe is a saving clause in the use of such gold, and that is the perfect malleting of the gold after its insertion in the cavity. It is a harder gold when once properly inserted. It is difficult to cut down, and when you attempt to finish one of these fillings you will find it a difficult problem, if you build it up too high, to restore the proper contour. It cuts more like iron than ordinary gold.

Dr. S. J. Renz. This gold is absolutely pure, will stand the greatest amount of heat to anneal it thoroughly, thus securing cohesion, which means the adhesion of two similar substances by heat.

Dr. Hetrick placed a filling in a cavity in the anterior approximal lower first molar, occupying nearly one-third of the occlusal surface of the tooth. The cavity was excavated, and from the time the first pellet of gold was placed in the tooth until the operation was complete he occupied thirty-two minutes of time. We calculated that there was at least seven minutes of interruption, which would make twenty-five minutes required to place in that amount of gold, which occupied at least one-third of the coronal surface. The cavity was prepared without any apparent groove except in the palatal wall, where a slight depression was made. The filling was placed with a steel matrix placed in position, which was removed and the filling contoured out. The gentlemen who witnessed the operation tried a Gem wheel upon it, and the density of the filling was amazing. The last portion of Dr. Hetrick's condensing was a revelation to me; it was done with burnished pluggers. After he had condensed thoroughly, he took pluggers that were perfectly smooth and went all over the surface, he then took a foot-burnisher to condense further and disclose any pits or depressions. He then showed how easy it was to attach to the palatal margin, this gold, and it was remarkable how readily it could be done.

Dr. Hetrick. In closing this discussion, I want to say there might be a misapprehension in regard to some other forms of gold. I do not claim that there is such a great saving of time in the use of Vernon's gold over other forms of foil gold, but there is some advantage in time. Just as good a filling can be put in with Moss fiber gold as can be put in with Vernon's gold, but it takes a little more time.

I want to say however, that I hope I will never find it necessary to go back to the use of foil. In the hurry and bustle of this age in which we live, the thing that saves the wear and tear on the nervous system of both dentist an patient is the thing we must take into consideration; and we must never lose sight of the personal equation also in the matter of the use of these filling materials. What I might be able to use to the best advantage, you might not be able to so use and therefore you should not adopt it if you can not do your best work with it.

I want to emphasize what Dr. Noble said in regard to filling small cavities. If you cut your pellets of gold smaller than the cavity, you will not incur the danger of "bridging" as Dr. Noble terms it. You would not have as much danger in using Moss fiber, because that is a more tractable kind of gold, and you would naturally expect to drive it ahead of your plugger. I feel that I can in justice speak a good word for both kinds of gold, but I am more enthusiastic over this particular form of gold which is my subject to-day. In closing, I want to reiterate again the caution of carefulness in in annealing, thoroughness in condensing and malleting and then you will find that you don't need to leave the rubber dam on to finish your gold filling. I think of all the barbarous things a dentist ever did, was to finish a gold filling with the rubber dam on.

Dr. Ambrose. A more barbarous method is to take it off.

Dr. Hetrick. No, I beg your pardon. If you will let me fill a tooth for you with Vernon's gold and finish it for you, you will take back that statement.

Dr. Ambrose. I was not speaking about Vernon's gold when I made that statement.

Dr. Hetrick. You can finish it just as well as you can with the dam on.

Dr. Ambrose. Between the teeth, how would you get along when it is wet.

Dr. Hetrick. Just as well as you would if it was dry?

Dr. Root. Work with wet sand-paper?

Dr. Hetrick. You get a better polish with wet sand-paper.

Dr. Root. You are not polishing, you are cutting down.

Dr. Hetrick. You don't cut it down with sand-paper, you cut it down with a little instrument that goes between. I use one of Rhein's trimmers. I do not see any reason for leaving the rubber dam on, because if you get a disc wet, put on another one. Some say you will cut the gums and make them bleed and all that sort of thing, but you need not do that if you will use care. It has been twenty years since I finished any gold filling with a rubber dam on.

Dr. Ambrose. There are some cavities that run up under the gum, and you draw a strip through there and cause more pain than the pressure of the clamp will cause. There is nothing that I can get through there that will polish properly except the strip.

Dr. Hetrick. I finish with those trimmers. I could not convince you of the correctness of my position except by an actual demonstration. That is the method I have adopted for years in finishing fillings, and I do it that way because it secures greater ease for the patient, not because it was easier for me. I will tell you, gentlemen, we do not want to lose sight of the fact that it is our duty to save the patient every particle of discomfort we can. If you can finish a filling easier with a rubber dam on, use it. Use instruments that you can handle the best. I can finish them with less discomfort to the patient and with less trouble to myself. If you use a matrix, there will be very little finishing required where the cavity goes under the cervical margin of the gum.

Nearly all of the anterior teeth I fill, I cut from the lingual side. Many times you can put in a thin strip of steel, using a wedge of wood or a separator to hold the matrix, and you can fill up above the gum line with the matrix in place, then take it off and finish condensing and you have very little polishing to do. I do this and believe in it thoroughly, because it saves discomfort to the patient.

I believe in conserving the energies of the patient. We must never lose sight of that, but, of course, must never do it at the expense of thoroughness of the operation. I did hurt these gentlemen some to-day. I would not have done them proper service had I not hurt them a little, but I do not believe any of them will say, because I took the rubber dam off and finished the fillings in that manner, that I gave them as much pain as I would had I done it the other way, or that I was unnecessarily rough in my manipulation.

In using any form of gold, if we take for our motto, that good enough never will do, but that each succeeding step in building up the character of our professional attainments must be a step in advance, more perfect adaptation, a better conservation of the energy of both patient and dentist, a more thorough understanding of the materials that we use and of the functions they are to perform in the mouth, and a determination to employ thoroughness in every operation, we will from day to day take a step higher in the attainment of a more perfect professionalism.

Dr. Cone. I want to say just another word in favor of Dr. Hetrick's method of finishing fillings. I believe it is a good way to finish them, for in those large fillings, there was no heat generated that would cause any aching of the tooth, and I have had some small fillings put in before in which I was caused intense pain when the strip was drawn through. By thoroughly moistening and keeping water on the tooth there is no high heat generated with the disc or with the strip.

